# miR-21a-5p Contributes to Porcine Hemagglutinating Encephalomyelitis Virus Proliferation via Targeting CASK-Interactive Protein1 *In vivo* and *vitro*

**DOI:** 10.3389/fmicb.2017.00304

**Published:** 2017-03-01

**Authors:** Xiaoling Lv, Kui Zhao, Yungang Lan, Zi Li, Ning Ding, Jingjing Su, Huijun Lu, Deguang Song, Feng Gao, Wenqi He

**Affiliations:** ^1^Key Laboratory of Zoonosis, Ministry of Education, College of Veterinary Medicine, Jilin UniversityChangchun, China; ^2^Key Laboratory of Zoonosis, Ministry of Education, Institute of Zoonosis, Jilin UniversityChangchun, China

**Keywords:** porcine hemagglutinating encephalomyelitis virus, coronavirus, miR-21a-5p, Caskin1, neurologic damage

## Abstract

Porcine hemagglutinating encephalomyelitis virus (PHEV) is a highly neurovirulent coronavirus that can cause nervous symptoms in piglets with muscle tremors, hind limb paralysis, and nystagmus. Whether some factors affect virus replication and proliferation had not been fully understood in the course of nerve damage caused by PHEV infection. In recent years, some reports suggested that miRNA might play a key regulatory role in viral infection. In this study, we found the miR-21a-5p is notably up-regulated in the brains of mice and N2a cells infected with PHEV, and it down-regulated the expression of CASK-interactive protein1 (Caskin1) by directly targeting the 3′-UTR of Caskin1 using a Dual-Luciferase reporter assay. The over-expression of miR-21a-5p or Caskin1 knockdown in the host significantly contributes to PHEV proliferation. Conversely, the silencing of miR-21a-5p by miR-21a-5p inhibitors suppressed the virus proliferation. Taken together, our results indicate that Caskin1 is the direct target gene of miR-21a-5p, and it is advantageous to virus proliferation by down-regulating Caskin1. These findings may help in the development of strategies for therapeutic applications.

## Introduction

Porcine hemagglutinating encephalomyelitis is an acute and highly contagious disease in pigs, mainly affecting piglets within 3 weeks of age, causing vomiting and wasting disease, as well as obvious neurological symptoms. The disease has not yet effective prevention and treatment measures currently. (Chen et al., 2011; Gao et al., 2011; Li Z. et al., 2016). The mortality rate ranges from 20 to 100% (Lan et al., 2012, 2013). This disease is caused by a member of the *Coronaviridae* family, which be known as porcine hemagglutinating encephalomyelitis virus (PHEV) (Dong et al., 2014); it is an enveloped virus containing a non-segmented, single-stranded, positive-sense RNA genome of approximately 30 kb. Pigs are the natural host of PHEV, but have been adapted to replicate in mouse and mouse neuroblastoma N2a cells (Chen et al., 2011). PHEV is a highly neurovirulent virus that spreads to the central nervous system via peripheral nerves (Dong et al., 2015), but the mechanism of induction of nerve injury is unclear. It is of great scientific interest to study the pathogenesis of PHEV from the point of view of virus infection and host protein interaction for the development of new antiviral drugs and treatment programs.

microRNAs (miRNAs) are non-coding ssRNAs that are 19–25 nt in length and post-transcriptionally regulate the expression of multiple genes by combining with the 3′-untranslated region (UTR) of their target messenger RNAs and thus become crucial regulators in complex gene regulatory networks (He and Hannon, 2004; Varnholt, 2008; Chi et al., 2009; Mallick et al., 2009). Accumulating evidence indicates that miRNAs play a important role in the infection of coronavirus and the neurovirulent virus (Hasan et al., 2014; Lai et al., 2014; Song et al., 2015; Zhu et al., 2015; Piedade and Azevedo-Pereira, 2016). For example, during SARS coronavirus infection process, miR-17^∗^, mir-574-5p, and miR-214, were up-regulated, and miR-98 and miR-223 were down regulated. Among these miRNAs, miR-17^∗^, mir-574-5p inhibited the replication of SARS coronavirus, whereas miR-214 contribute to immune escape of the bronchial alveolar stem cells (BASC) (Mallick et al., 2009). miR-15b modulates the inflammatory response during JEV infection by negatively regulating RNF125 expression (Zhu et al., 2015). Our previous research revealed that miR-21a-5p, which is highly homologous with miR-21, was significantly increased in the process of PHEV infection by a DNA microarray analysis (Data are not published).

miR-21 is a multifaceted microRNA regulating the expression of target genes involved in several cellular programs, such as cell proliferation, migration, invasion, and metastasis (Krichevsky and Gabriely, 2009; Zhang et al., 2013). The regulatory role of miR-21 in process of viral infection was confirmed by a number of studies and can be used as a target for the treatment of viral diseases. For example, in the murine coxsackievirus B3 (CVB3)-induced myocarditis model, the expression of miR-21 was significantly reduced. The recovery of miR-21 expression significantly relieved CVB3-induced myocarditis as shown by an increased body weight, a reduced myocardial injury, a lowered myocarditis score and an increased survival rate. Further study showed that miR-21 protects against myocardial apoptosis by specifically inhibiting the expression of its target programmed cell death 4 (PDCD4). These data proved miR-21 might be a novel target for the treatment of CVB3 infection and other apoptosis-mediated cardiovascular diseases (He et al., 2013).

In this study, we sought to investigate the regulatory role of miR-21 in PHEV proliferation and provide theoretical basis for the development of a new therapeutic regimen for PHEV infection.

## Materials and Methods

### Cells, Virus, and Mice

Mouse neuroblastoma N2a cells (N2a) and a human cervical carcinoma cell line (Hela) were obtained from Professor Xia (Military Veterinary Institute, Academy of Military Medical Sciences, Changchun, China). N2a cells and Hela cells were maintained in Dulbecco’s Modified Eagle’s medium (DMEM) (Gibco, USA) containing 10% fetal calf serum, 1% streptomycin, 1% penicillin, and were incubated at 37°C in a wetted chamber supplemented with 5% CO_2_. The PHEV strain HEV 67N (GenBank: AY048917) was propagated in N2a cells. BALB/c mice (3 weeks old) were obtained from the Laboratory Animal Centre, Jilin University.

### The Choice of Housekeeping Genes

Generally, U6 and GADPH are expressed at relatively constant levels in normal and pathological conditions. These genes may be used as housekeeping genes in brain damage (Chi et al., 2014; Alarcon et al., 2015; Li G. et al., 2016; Shen et al., 2016). There are no significant differences in the expression of U6 and GADPH in the gene expression patterns in the cerebral cortex of mice infected with PHEV detected using microarray in our previous study (Lan et al., 2014), so we take them as internal reference genes for the relative quantification of other genes in this study.

### RT-PCR for miRNA and mRNA Expression

miRNA-enriched total RNA was extracted from N2a cells, Hela cells and brain tissues of mice infected with PHEV using a miRNApure Mini Kit (cwbio, China). To analyze Caskin1 (GenBank: NM_027937.2) mRNA expression, RNA was extracted using Trizol, tissue: 50–100 mg tissue/ml, cell: 10 cm^3^/ml. The concentration of RNA was detected by spectrophotometer (Thermo Scientific). RNA was reverse transcribed into cDNA by using the reverse transcription kit (Takara, Japan). The quantification of miRNAs was performed using the Bulge-Loop^TM^ miRNA qRT-PCR Primer Set (RiboBio, China). miR-21a-5p and Caskin1 expressions were determined by RT-PCR using SYBR Green Master Mix kit as described previously (Shen et al., 2016). The relative expression was analyzed using the 2^-ΔΔCT^ method. U6 and GAPDH were used for normalization of miR-21a-5p and Caskin1 expression, respectively (Shen et al., 2016). The cycle conditions and the system for PCR were set according to the manufacturer’s protocol. U6 and mmu-mir-21a-5p primers were purchased from RiboBio. The primers for Caskin1 and GAPDH were designed as follows: mouse Caskin1 sense primer, 5′-GTGGGTCGGAGCCATTCA-3′; anti-sense primer, 5′-GCCGAGCTGGAGCGTTT-3′; mouse GAPDH sense primer, 5′-CTCAACTACATGGTCTACATGTTC-3′; anti-sense primer, 5′-ATTTGATGTTAGTGGGGTCTCGCTC-3′; HEV sense primer, 5′-AGCGATGAGGCTATTCCGACTA-3′; and anti-sense primer, 5′-TTGCCAGAATTGGCTCTACTACG-3′. The PCR reaction system was 20 μL and reaction conditions: pre-degeneration at 95°C for 3 min, denaturation at 95°C for 30 s, annealing at 60°C for 30 s, extension at 72°C for 30 s with a total of 40 cycles. The amplification efficiency of PCR was detected (Supplementary Data Sheet 1).

### Cloning of the 3′-UTR/Caskin1 Dual-Luciferase Reporter Construct

The miR-21a-5p targets were predicted by TargetScan, Microcosm and Miranda. Caskin1 was used as the research object. The 3′-UTR of mouse Caskin1 gene, containing the putative miR-21a-5p binding site (Caskin1-WT-UTR), was amplified from mouse genomic DNA by PCR. The 3′-UTR of mouse Caskin1 gene, containing a mutant miR-21 binding side (Caskin1-MUT-UTR), was created by overlap extension of PCR. The Caskin1-WT-UTR and the Caskin1-MUT-UTR were subcloned into pmirGLO Dual-Luciferase miRNA Target Expression Vector (Promega, USA) at the Sac I and Xhol site (Caskin1-WT and Caskin1-MUT, respectively). The primers for the 3′-UTR of Caskin1 were as follows: Caskin1-WT-UTR sense primer, 5′-GGCACAGCACAAGGGACA-3′; anti-sense primer, 5′-CAGTAAGAGCAAGGCACATCC-3′; Caskin1-MUT-UTR, primer1, 5′-GGCACAGCACAAGGGACA-3′; primer2, 5′-TACTCCTTGATAGCGCATATTCAGGGGTGCAGTGGGGCG-3′ primer3; 5′-CGCCCCACTGCACCCCAGAATATGCGCTATCAAGAGTA-3′ primer4; and 5′-CAGTAAGAGCAAGGCACATCC-3′.

### Cell Transfection

Hela cells were plated in six-well plates at a density of 3 × 10^5^ cells/well in DMEM containing 2% fetal bovine serum and were grown overnight. X-tremeGENE HP DNA Transfection Reagent (Roche, Sweden) was used to co-transfected Hela cells with 50 nM miR-21a-5p mimic or 100 nM inhibitor or their respective non-targeting negative control oligonucleotides (RiboBio) and 2 μg of Caskin1-WT or Caskin1-MUT. The empty plasmid pmirGLO group was used for the negative control, and non-transfected Hela cells were used as the blank control. After 48 h of transfection, luciferase activity was detected after transfecting 48 h by using a dual luciferase reporter assay system (Promega). Renilla luciferase activity was used for normalization. N2a cells (3 × 10^5^ cells per well) were seeded into six-well culture plates, incubated overnight and transfected with 50 nM of the miR-21a-5p mimics or 100 nM of the miR-21a-5p inhibitor or the Caskin1 siRNAs or the siRNA NC using X-tremeGENE HP DNA Transfection Reagent (Roche). Their respective non-targeting negative control oligonucleotides and a scrambled siRNA (siNC) were used as the negative controls. The cells were inoculated with virus 12 h after the transfection. All the transfection experiments were repeated at least three times.

### Western Blotting Analysis

The cells in 6-well plates or brain tissues were washed once with phosphate buffer saline (PBS), followed by lysis using a Radio Immunoprecipitation Assay (RIPA) Lysis Buffer and a Phenylmethanesulfonyl fluoride protease inhibitor (Beyotime) on ice for 30 min. The concentration of protein was determined by the BCA Protein Assay kit (Pierce). The protein samples (50 mg/lane) were separated using a 10% polyacrylamide gels and were transferred to 0.22 μm polyvinylidene fluoride membranes using the Bio-Rad wet transfer system. After blocking overnight at 4°C with 5% non-fat dry milk in PBS, the membranes were probed with antibodies against Caskin1 (Synaptic Systems, Göttingen, 1:2000), β-actin (Proteintech, USA, 1:2000) and PHEV (a laboratory-prepared polyclonal antibody to PHEV, 1:500) with an overnight incubation at 4°C. Next, the membranes were washed with PBS containing tween-20 (PBST) four times and were incubated with horseradish peroxidase-linked secondary anti-rabbit or anti-mouse IgG antibodies (Proteintech) for 1 h at 37°C. After washing with PBST, the signal was visualized using an ECL detection kit (Proteintech). β-actin was used as a loading control.

### TCID_50_ Analysis

To determine the TCID_50_ of the virus culture collected at different passages, the cell culture supernatants were serially diluted from 10^-1^ to 10^-8^, and 100 μL of the diluted virus was inoculated onto the N2a cells in each well of the 96-well culture plates with eight wells for each dilution. The plates were incubated for 3 days at 37°C in 5% CO2 and were scored for a cytopathic effect. The infectious titer was calculated by the Reed and Muench method (Biacchesi et al., 2005).

### PHEV Infection and miR-21a-5p Antagomir Administration

The mice were randomly divided into four groups, six mice in each group, as follows: group 1 was the control group; group 2 was the PHEV-infected and PBS group (PBS); group 3 was the PHEV-infected and antagomir control group (NC); and group 4 was the PHEV-infected and miR-21a-5p antagomir treated group (antagomir). The miR-21a-5p antagomir used in this study contains chemically modified single-stranded RNA molecules which could prevent the complementary pairing of miRNA and its target gene mRNA through the combination of strong competitive with the mature miRNA *in vivo* were purchased from RiboBio. The mice in the antagomir group were injected intraperitoneally with 2 nmol antagomir per mouse. The mice in the other groups were injected with the same volume of control solution or not. The brain tissues were analyzed 24 h after an intracerebral injection to study the expression of miR-21a-5p and Caskin1 by RT-PCR or Western blotting. After 24 h of injection, mice were inoculated intranasally with 100 ml of PHEV solution (TCID_50_ = 10^-4.5^/0.1 ml). The brain tissues were analyzed 5 days after the inoculation with PHEV to analyze the expression of miR-21a-5p and Caskin1 and viral RNA by qRT-PCR or Western blotting. The weight of the mice was measured every day. The permission to work with laboratory animals was obtained from the Animal Welfare Ethical Committee of the College of Veterinary Medicine, Jilin University, China.

### Indirect Immunofluorescence

After inoculation with PHEV, the mice were sacrificed, and the brain tissues were cut into frozen-sections. The frozen-sections or the cells grown in 6-well plates, after transfection, were washed with PBS, fixed with 4% paraformaldehyde for 15 min at room temperature, permeabilized with 0.1% Triton X-100 for immunofluorescence for 15 min at room temperature and were blocked with 5% non-fat milk powder for 1 h at 37°C before being washed with PBS and incubated overnight at 4°C with a PHEV polyclonal antibody. After washed with PBS three times, the (FITC)-conjugated Affinipure Goat Anti-Mouse IgG (H+L) secondary antibodies (Proteintech) were incubated with PBS at 37°C 1 h. Hoechst was used to stain the nuclei. After washed with PBS three times, the coverslips were mounted onto glass with Antifade Solution (Solarbio) before visualization on a confocal microscope.

### Statistical Analysis

Values are presented as an arithmetic mean ± standard error. All data were analyzed by SPSS 17.0 software (Chicago, USA). Histogram was carried out with GraphPad Prism 5.0 software (San Diego, CA, USA). Western blot pictures were analyzed by Tanon Gis software (Shanghai, China). Fluorescence intensity was analyzed by ImageJ software (National Institutes of Health, USA). All results were considered statistically significant at the *p*-values < 0.05 level.

## Results

### miR-21a-5p Up-Regulation during the PHEV Infection Process

**FIGURE 1:**
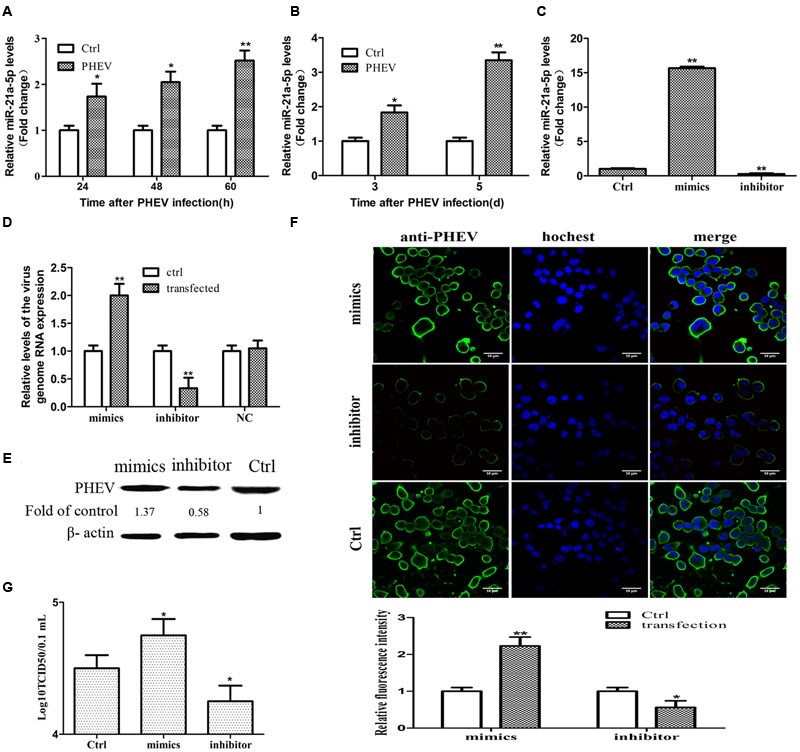
**miR-21a-5p was up-regulated during the porcine hemagglutinating encephalomyelitis virus (PHEV) replication process and promoted PHEV replication. (A)** N2a cells were infected with or without PHEV for different times as indicated. The expression of miR-21a-5p was measured by RT-PCR. **(B)** miR-21a-5p expression in the BALB/c mice brain after infecting with PHEV as determined by RT-PCR. **(C)** The expression of miR-21a-5p in the N2a cells after transfection with the indicated mimics or inhibitors of the indicated miR-21a-5p. **(D)** The levels of the virus genome RNA after transfection with the miR-21a-5p mimics or inhibitor. **(E)** A Western blot was performed to examine the expression of the PHEV protein. **(F)** IFA was performed to examine the expression of PHEV. **(G)** The PHEV titers in the N2a cell supernatants of the N2a cells after transfection with the miR-21a-5p mimics or inhibitor. All of the data are representative of at least three independent experiments. ^∗^*P* < 0.05, ^∗∗^*P* < 0.01 vs. normal controls.

To determine the differentiated expression of miR-21a-5p during the PHEV infection process, we collected the N2a cells after infection for 24, 48, and 60 h, and the mouse brain tissue was infected for 3 and 5 days prior to the RT-PCR. The results revealed that the relatively expression level of miR-21a-5p was significantly higher after infection than in the control (**Figures 1A,B**). Thus, we speculate that miR-21 might play a role in the process of viral infection.

### miR-21a-5p Promotes PHEV Replication *In vitro*

To determine whether mir-21a-5p has effects on PHEV replication, we tested the effect of upregulating or blocking miR-21a-5p on PHEV replication in N2a cells. To figure out the efficacy of the miR-21a-5p mimics and the inhibitor, the N2a cells were transfected with the miR-21a-5p inhibitor or the miR-21a-5p mimics for 24 h, and the expression level of miR-21a-5p was analyzed. A significant increase or decrease was observed in the miR-21a-5p level in the N2a cells transfected with the miR-21a-5p mimics or the miR-21a-5p inhibitor, respectively, compared to the cells transfected with the negative control (**Figure 1C**). The N2a cells were transfected with the mimics or the miR-21a-5p inhibitor (50 or 100 nM), followed by infection with PHEV. The cells were collected 24 h post-infection to determine the viral propagation. Among the RT-PCR, Western blotting, IFA and TCID50 results, the overexpression of miR-21a-5p significantly increased the progeny of PHEV production, and conversely, the transfection of the miR-21a-5p inhibitor demonstrated the opposite effects (**Figures 1D–G**). These data suggest that miR-21a-5p induction contributes to PHEV replication.

### The Prediction of miR-21 Target Genes

To characterize the molecular components of miR-21a-5p activity in facilitating PHEV replication, we next predicted miR-21a-5p targets using bioinformatics prediction software. TargetScan predicted 210, MicroCosm predicted 836 and miRanda predicted 4990 target genes. Of these, 203 target genes were predicted by all three systems. Then, the target genes were functionally analyzed. We found that the target genes were involved in a variety of physiological processes, such as cell differentiation, proliferation, apoptosis, and synaptic function (Supplementary Data Sheet 2). Part of results was demonstrated in **Table 1**. Caskin1, a newly discovered post-synaptic density protein in mammalian neurons, was used as the research object.

**Table 1:** The result of the miR-21a-5p target gene prediction.

mir base	Matuacc	mirna_name	Gene symbol	Gene description	Score
mmu-miR-21a-5p	MIMAT0000530	mmu-miR-21	Fnip1	Folliculin interacting protein 1	-0.25
mmu-miR-21a-5p	MIMAT0000530	mmu-miR-21	Pcbp1	Poly(rC) binding protein 1	-0.25
mmu-miR-21a-5p	MIMAT0000530	mmu-miR-21	Tmem170	Transmembrane protein 170	-0.24
mmu-miR-21a-5p	MIMAT0000530	mmu-miR-21	Caskin1	CASK interacting protein 1	-0.23
mmu-miR-21a-5p	MIMAT0000530	mmu-miR-21	Spg20	Spastic paraplegia 20, spartin (Troyer syndrome) homolog (human)	-0.23
mmu-miR-21a-5p	MIMAT0000530	mmu-miR-21	Chd7	Chromodomain helicase DNA binding protein 7	-0.23
mmu-miR-21a-5p	MIMAT0000530	mmu-miR-21	Rpa2	Replication protein A2	-0.23
mmu-miR-21a-5p	MIMAT0000530	mmu-miR-21	Klhdc5	Kelch domain containing 5	-0.23
mmu-miR-21a-5p	MIMAT0000530	mmu-miR-21	Chic1	Cysteine-rich hydrophobic domain 1	-0.23
mmu-miR-21a-5p	MIMAT0000530	mmu-miR-21	Jag1	Jagged 1	-0.22

### miR-21a-5p Modulates Caskin1 Expression in PHEV-Infected N2a Cells

**FIGURE 2:**
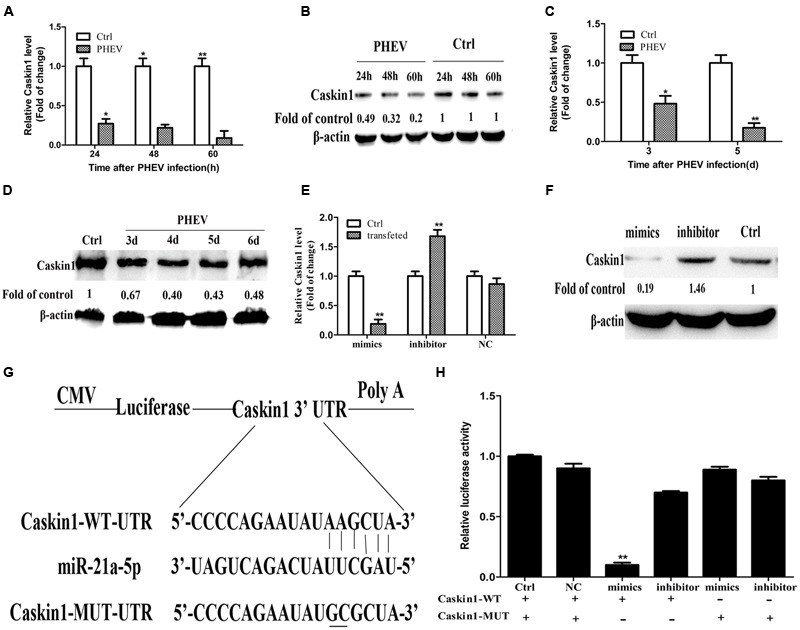
**miR-21a-5p directly regulates Caskin1 expression by targeting the 3′-UTR of Caskin1. (A)** Caskin1 mRNA expression in the N2a cells after infecting with PHEV by RT-PCR. **(B)** The expression of Caskin1 in the N2a cells after infecting with PHEV by Western blot. **(C)** Caskin1 mRNA expression in the BALB/c mice brain tissue after infecting with PHEV by RT-PCR. **(D)** The expression of Caskin1 in the BALB/c mice brain tissue after infecting with PHEV by Western blot. **(E,F)** The expression of Caskin1 after transfection with the miR-21a-5p mimics or inhibitor by using RT-PCR **(E)** or Western blot **(F)**. **(G)** The Dual-luciferase reporter construct containing the wild type or mutant 3′-UTR of Caskin1. Caskin1-WT-UTR, sequence of the putative miR-21 binding site; Caskin1-MUT-UTR, sequence of the mutant miR-21 binding site. **(H)** Dual-luciferase reporter activity in Hela cells 48 h post-transfection. All of the data are representative of at least three independent experiments. ^∗^*P* < 0.05, ^∗∗^*P* < 0.01 vs. normal controls.

The time-dependent expression pattern of Caskin1 mRNA and protein in the N2a cells and mouse brain tissue following PHEV infection was studied. A significant down-regulation in Caskin1 mRNA and protein expression at 24, 48, and 60-h post-PHEV infection was observed (**Figures 2A,B**). The results of the brain tissue detection in the mice were consistent with the above findings (**Figures 2C,D**). The mRNA and protein expression of Caskin1 also were determined after transfecting with the miR-21a-5p mimics which could be over-expressed miR-21a-5p. The expression of Caskin1 was significantly decreased after transfection with miR-21a-5p mimics (**Figures 2E,F**). Furthermore, the expression of Caskin1 in N2a cells following transfection with the miR-21a-5p inhibitor was analyzed. Inhibition the expression of miR-21a-5p caused the enhanced expression of Caskin1 mRNA and protein (**Figures 2E,F**). It was thus evident from the results that miR-21a-5p modulates Caskin1 expression.

### miR-21a-5p Directly Regulates Caskin1 Expression by Targeting the 3′-UTR of Caskin1

To test whether miR-21a-5p directly regulates the expression of Caskin1 in the process of the PHEV infection, we prepared a Dual-Luciferase miRNA Target Expression Vector by binding the 3′-UTR of mouse Caskin1 which containing an exact match to miR-21a-5p target sequence (Caskin1-WT-UTR) (**Figure 2G**). We also created a Dual-luciferase miRNA Target Expression Vector by binding the 3′-UTR of mouse Caskin1 which containing a mismatched version of miR-21a-5p target side (Caskin1-MUT-UTR) (**Figure 2G**) as control. Co-transfection of the Dual-luciferase miRNA Target Expression Vector containing the Caskin1-WT-UTR (Caskin1-WT) with the miR-21a-5p mimics in the Hela cells resulted in an approximate 90% loss of Dual-luciferase reporter expression compared with the control (**Figure 2H**). However, the Dual-luciferase expression was not affected by the co-transfection with the miR-21a-5p mimics when the Caskin1-WT-UTR was replaced with the Caskin1-MUT-UTR in the Dual-luciferase reporter system (**Figure 2H**). Similarly, luciferase activity was significantly increased when the Hela cells were transfected with the miR-21a-5p inhibitor to inhibit the endogenous miR-21 levels (**Figure 2H**). Taken together, these results indicate that miR-21a-5p negatively regulates Caskin1 expression in the PHEV infection process by straightly binding the 3′-UTR of the Caskin1 gene.

### miR-21a-5p Promotes PHEV Replication by Targeting Caskin1 in the N2a Cells

**FIGURE 3:**
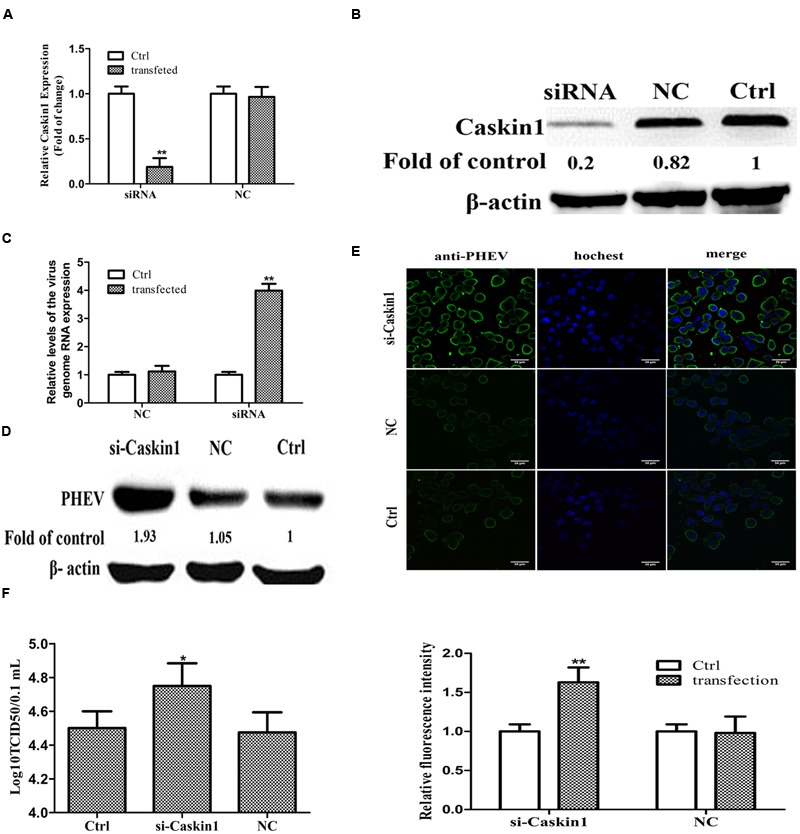
**miR-21a-5p promotes PHEV replication by targeting Caskin1 in N2a cells. (A)** Caskin1 mRNA levels in N2a cells 48 h post-transfection with siRNAs. **(B)** Caskin1 protein levels in N2a cells 48 h post-transfection with Caskin1 siRNA. **(C)** The levels of the viral genome RNA after transfecting with Caskin1 siRNA. **(D)** Western blotting was performed to examine the expression of PHEV protein. **(E)** IFA was performed to examine the expression of PHEV. **(F)** Viral titers from N2a cells transfected with Caskin1 siRNA. All of the data are representative of at least three independent experiments. ^∗^*P* < 0.05, ^∗∗^*P* < 0.01 vs. normal controls.

Then we tested whether the expression of Caskin1 had an effect on PHEV replication. First, the expression of endogenous Caskin1 was reduced by transfecting Caskin1 siRNA in the N2a cells. The results showed that more than 80% Caskin1 mRNA and protein levels was silenced in the N2a cells (**Figures 3A,B**). Then, we examined the effect of reduced Caskin1 expression on PHEV replication. PHEV replication was significantly increased in the cells after reducing the expression of Caskin1 compared to the cells transfecting with a negative-control siRNA (**Figures 3C–E**). Cells in which Caskin1 was silenced had a significant increasing trend in PHEV titers compared to the control cells (**Figure 3F**). In conclusion, these results indicate that Caskin1 is a conditioning factor of PHEV replication and that PHEV makes use of miR-21a-5p induction to decrease Caskin1 levels and conducive to its replication.

### miR-21a-5p Antagomir Treatment Reduces Symptoms in PHEV-Infected Mice

**FIGURE 4:**
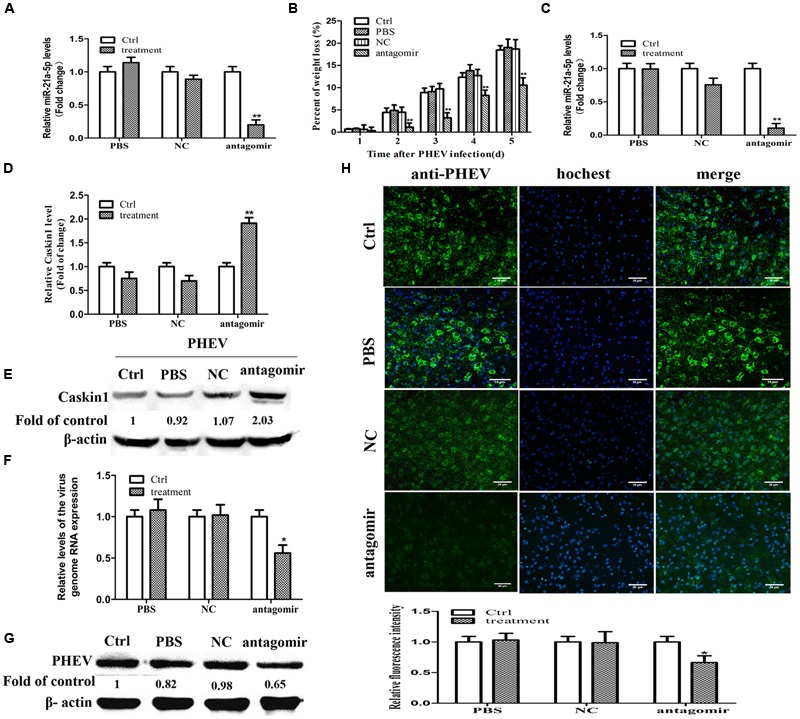
**miR-21a-5p antagomir treatment reduces symptoms in PHEV-infected mice. (A)** The expression of miR-21a-5p in brain tissue 24 h after injection. **(B)** Weight loss rate in the mice after treatment during PHEV infection. **(C)** The expression of miR-21a-5p in brain tissue 5 days after PHEV inoculation. **(D)** The expression of Caskin1 mRNA in brain tissue 5 days after PHEV inoculation. **(E)** The expression of Caskin1 protein in brain tissue 5 days after PHEV inoculation. **(F)** The relative expression of the viral RNA in brain tissue after treatment. **(G)** The expression of PHEV protein in brain tissue after treatment. **(H)** IFA was performed to examine the expression of PHEV in brain tissue after treatment. All of the data are representative of at least three independent experiments. ^∗^*P* < 0.05, ^∗∗^*P* < 0.01 vs. normal controls.

To determine whether miR-21a-5p inhibition was possible in normal mice *in vivo*, the miR-21a-5p antagomir was injected intracerebrally three times, at 3-day intervals (at days 0, 3, and 6). We detected miR-21a-5p expression in the brain tissue of the different groups of mice. In the miR-21a-5p antagomir group, the miR-21a-5p expression levels were significantly down-regulated 24 h after injection compared with the control (**Figure 4A**). The result indicated that the miR-21a-5p antagomir efficiently entered into the mouse brain tissue, resulting in the deletion of miR-21a-5p in the mouse brain tissue.

On day 5 post-infection, the control group, the PBS group, and the NC group mice exhibited typical symptoms of PHE that included generalized muscle tremors and hyperesthesia. However, the antagomir group did not have these typical symptoms. At 7 dpi, the antagomir group appeared to have the typical symptoms. Unfortunately, all of the mice in those groups eventually died. In contrast, the antagomir group of mice survived more than 2 days. In addition, compared with other groups, the antagomir group of mice did not have an obvious weight loss (**Figure 4B**). At 5 dpi, the mice were sacrificed, and the brain samples were collected and processed for subsequent experiments. The results were similar to those *in vitro*, it has a negative relationship between the expression patterns of miR-21a-5p and its target Caskin1 in the brain tissues from the PHEV-infected mice, and the higher expression of miR-21a-5p was associated with a low-level of Caskin1 (**Figures 4C–E**). In the PHEV-infected mice, the treatment with the miR-21a-5p antagomir caused a significant reduction in the miR-21a-5p expression and rescued the alterations in the Caskin1 levels (**Figures 4C–E**).

We used RT-PCR, IFA, and Western blotting to determine the effects of the miR-21a-5p antagomir on the viral proliferation after injection. The expression of viral RNA and protein was down-regulated after injecting the miR-21a-5p antagomir (**Figures 4F,G**). The results of the IFA indicated that the miR-21a-5p antagomir had a certain effect on the proliferation of the virus (**Figure 4H**). These findings affirm that the miR-21a-5p antagomir inhibits viral proliferation by up-regulating Caskin1 and has a therapeutic effect on animals.

## Discussion

Many studies show that miRNA and viral infection are closely related processes, such as Epstein Barr virus, herpes virus and some reverse transcription viruses because virus-encoded miRNAs can regulate host cell endogenous miRNA expression (Pfeffer et al., 2004). The host cell’s endogenous miRNA inhibits the replication of the virus, and there could also be a virus that facilitates viral replication or regulates cellular immune function and so on (Sarkies et al., 2013). For example, the host cell miR-145 negatively regulates replication of the oncolytic herpes simplex virus-1 by targeting AP27i145 (Li et al., 2013), and in influenza virus infection, multiple proteases play a key role in the host cell miRNA regulation of these proteases, which permits convenient influenza virus replication (O’Connor et al., 2014). In addition, miR-29b in the JEV-infected mouse microglial cell line was up-regulated during JEV-induced microglial activation, and miR-29b plays a role of the pro-inflammatory response. The mechanisms of its action is mediated by inhibiting the anti-inflammatory protein, TNFAIP3, resulting in the continuous activation of NF-κB and followed by pro-inflammatory cytokine secretion (Li et al., 2013).

Multiple miRNAs modulate the virus infection process, but the roles of the miRNAs in the PHEV infection process are not fully understood. Neurotropic viruses such as JEV, human immunodeficiency virus 1, herpes simplex virus 1, and vesicular stomatitis virus could cause host cell miRNA expression changes during infection. The expression of miRNAs, which normally regulates viral replication, was upregulated by 1.5–4-fold (Ashraf et al., 2016). The previous research from our lab demonstrated that the change in the miR-21a-5p expression was obvious in the PHEV infection process, suggesting that miR-21a-5p might play a very important role in the process of virus infection. PHEV mainly causes obvious nerve injury, whereas miR-21 also plays an important role in nerve injury. For example, in traumatic brain injury, the expression of miR-21 was up-regulated 1.5-fold in the brain cortex and hippocampus and might affect the pathophysiology of traumatic brain injury (Redell et al., 2011). In this study, we found that miR-21a-5p expression in N2a cells was up-regulated after PHEV infection and increased up to 2.5-fold at 60 h. The expression of miR-21a-5p was also up-regulated in PHEV-infected mice and increased to 3.35-fold at 5 days. This indicates that the expression level of miR-21a-5p is significantly increased during PHEV-infected host, suggesting that miR-21a-5p may play a role in PHEV-induced neurotoxicity. In addition, the up-regulation of miR-21a-5p expression promotes viral proliferation, and the knock-down of the expression of miR-21a-5p reduces viral proliferation, suggesting that miR-21a-5p affects PHEV proliferation. Unlike other cells, nerve cells are very sensitive to injury, especially to some neurotropic virus infections, such as PHEV. Small changes in the amount of the viruses in the host neurons may cause significant changes in the course of disease. In spite of siRNA inhibition of Caskin1 showed a very modest inhibition of PHEV replication (twofold) in neuronal cell cultures and even less of an effect in mice, its impact on viral nerve injury may be very large. Therefore, we speculated that miR-21a-5p might play an important role in PHEV pathogenesis. Whether there are other targets of miR-21a-5p affect viral replication is unclear during PHEV infection.

miRNAs post-transcriptionally regulate the expression of multiple genes by binding to the 3′-UTR of their target messenger RNAs to play biological functions (Chi et al., 2009). To determine the mechanism of miR-21a-5p affecting virus proliferation, we predicted its target genes. There were many target genes of miR-21, such as PTEN, PDCD4, RECK, TPM1, TIMP-3, Maspin, and Sprouty (Spry-2, Spry-1) (Buscaglia and Li, 2011). These target genes were involved in the process of cell proliferation and apoptosis and so on. In this study, we choose Caskin1 as the target gene to be detected. Caskin1 is a brain-specific multi-domain scaffold protein that binds Lar and Dock through its different structural domains. In addition, Caskin1 plays a key role in motor axon targeting through interaction with the Lar-dependent signaling pathway (Weng et al., 2011). In the CNS, Caskin1 and dock have overlapping roles in axon outgrowth. Overall, these studies indicate Caskin1 is required for neuronal axon growth and guidance in the CNS. Together, these studies identify Caskin1 as a neuronal adaptor protein required for axon growth and guidance (Weng et al., 2011). Time dynamics research to the expression of miR-21a-5p and its target gene Caskin1 showed that an inverse relationship with respect to each other’s expression until the 24 h time point. The up-regulation of miR21a-5p after PHEV infection showed a sustained changes at 24, 48, and 60 h; however, Caskin1 mRNA and protein levels decreased 60 h post-infection compared to the 24 and 48 h time points. It is possible that miR-21a-5p reduces the expression of its target mRNA and protein (Caskin1). These results were consistent with the above *in vivo* experiment. However, further studies are required to resolve this issue of the differential kinetics of miR-21a-5p and its target. In this study, we used a luciferase reporter assay to evaluate the interaction of miR-21a-5p with the 3′-UTR of Caskin1. It was evident from the luciferase reporter assay that miR-21a-5p binds to this region of Caskin1 and suppresses its expression. The knock-down of the expression of Caskin1 in the N2a cells promotes virus proliferation. Taken together, our findings demonstrate that miR-21a-5p positively regulates PHEV replication by targeting Caskin1.

To study the role of miRNA *in vivo* and its influence on the viral diseases, the miRNA antagomir was used to reduce the miRNA concentration. For example, after the miR-19b-3p antagomir treatment, 40% of the JEV-infected mice became asymptomatic, and the expression of miR-19b-3p showed a reciprocal pattern with its target gene RNF11 in the JEV-infected mouse brain tissues. The miR-19b-3p antagomir inhibits cytokine secretion and activation of astrocytes and microglia, and reduces neurons damage in the JEV-infected mice (Ashraf et al., 2016). In this study, miR-21a-5p antagomir treatment delayed the onset of mice and delayed their weight loss, and the lifespan of the mice was extended for about 2 days. After the miR-21a-5p antagomir treatment, the virus multiplication decreased in the PHEV-infected mouse brain tissues. MiR-21a-5p exhibited a negative regulation expression profile with Caskin1 in the brain of PHEV-infected mouse, which further supports a functional interaction between the miRNA and mRNA *in vivo*. These findings indicate that the miR-21a-5p antagomir treatment reduces the symptoms in the PHEV-infected mice.

In this study, we identified a new mechanism regulating the proliferation of PHEV mediated by interaction between miR-21 and Caskin1, which may be exploited to reduce the proliferation of PHEV for therapeutic applications.

## Ethics Statement

All of the mouse experiments in this study were approved by the Animal Welfare Ethical Committee of the College of Veterinary Medicine, Jilin University, China (permission number 2012-CVM-12) and were performed in accordance with the guidelines of the Council for the International Organization of Medical Sciences on Animal Experimentation (World Health Organization, Geneva, Switzerland).

## Author Contributions

XL and WH conceived and designed the experiments. XL, KZ, YL, ZL ND, and JS performed the experiments. XL and FG analyzed the data. HL and DS contributed reagents, materials, and analysis tools. XL and WH wrote the manuscript. All of the authors read and approved the final manuscript.

## Conflict of Interest Statement

The authors declare that the research was conducted in the absence of any commercial or financial relationships that could be construed as a potential conflict of interest.
